# Exogenous ALA applied on different plant parts promotes tomato fruit quality and GABA synthesis

**DOI:** 10.3389/fnut.2024.1520634

**Published:** 2025-01-03

**Authors:** Peng Bai, Junwen Wang, Yongmei He, Junfang Feng, Juanli Li, Xingpan Shang, Yue Wu, Jihua Yu, Zhongqi Tang, Jianming Xie

**Affiliations:** ^1^College of Horticulture, Gansu Agricultural University, Lanzhou, China; ^2^State Key Laboratory of Aridland Crop Science, Gansu Agricultural University, Lanzhou, China

**Keywords:** tomato, 5-aminolevulinic acid, *γ*-aminobutyric acid, fruit quality, free amino acid

## Abstract

**Introduction:**

Tomato fruit are rich in *γ*-aminobutyric acid (GABA), which lowers blood pressure and improves sleep. An increase in GABA content is important for enhancing the nutritional quality of tomato fruit.

**Methods:**

To investigate the effects of 5-aminolevulinic acid (ALA) on fruit quality and GABA synthesis in greenhouse tomatoes, the tomato cultivar (*Solanum lycopersicum* cv. ‘184’) was used as an experimental material. During the fruit-setting period, root drenching with 0, 10, and 50 mg·L^−1^ ALA, foliar spraying with 0, 50, and 100 mg·L^−1^ ALA, and fruit surface spraying with 0, 100, and 200 mg·L^−1^ ALA were applied. The study investigated the application of exogenous ALA to different parts of the plant to determine the optimal ALA concentrations for each application site (10 mg·L^−1^ for root application, 100 mg·L^−1^ for foliar application, and 100 mg·L^−1^ for fruit surface application). Using the selected optimal ALA concentrations, tomatoes were used to study the effects of exogenous ALA application at different sites on fruit quality and GABA synthesis of greenhouse tomatoes.

**Results and discussion:**

The results demonstrated that exogenous ALA application to different parts of greenhouse-grown tomato plants substantially increased single-fruit weight by 42.37%–76.24%, soluble sugar content by 78.51%–94.52%, soluble solids by 9.09%–41.71%, soluble protein by 82.71%–241.05%, and ascorbic acid content by 1.31%–5.06% in mature tomatoes. And it reduced the organic acid content of the fruit by 12.81%–33.61%. Moreover, ALA applied at different parts of plants substantially enhanced the free amino acid content by 11.22%–16.50%, among them, umami amino acid content by 7.26%–20.13%. Besides, GABA content in mature tomato fruits was increased by 214.58%–433.32 across the different application parts. Exogenous ALA application at different sites regulates the activity of glutamate acid decarboxylase (GAD) and increases the content of glutamate for GABA synthesis pathway during tomato fruit development, thereby affecting the GABA content. In summary, exogenous ALA applied at different parts of tomato plants regulates the metabolism of amino acids and enhances the biosynthesis of GABA, which promotes the nutrient quality of the fruit.

## Introduction

Tomato (*Solanum lycopersicum*) is an important vegetable crop characterized by diverse fruit colors, unique flavors, and high nutritional value. The fruit is rich in GABA, sugars, organic acids, vitamins, amino acids, lycopene, and other essential minerals. These components have numerous benefits for human health, including blood pressure reduction, sleep quality improvement, cholesterol lowering, cancer prevention, and a decreased risk of cardiovascular and cerebrovascular diseases (1, 2).

The common nutrients in tomatoes include sugars, acids, vitamins, minerals, proteins, and carotenoids. They also contain various essential amino acids and unsaturated fatty acids necessary for the human body (3–6). The flavor quality of tomatoes depends on the content of soluble solids and volatile aromatic compounds. Among the soluble solids, sugar and organic acid components, and the sugar-acid ratio primarily influence the flavor of tomato fruit, such as sweet or sour flavor (7). Tomato ascorbic acid (Vc), an antioxidant, plays an indispensable role in plant responses to biotic and abiotic stress. Additionally, Vc is one of the most important nutrients in tomato fruit, helping to boost the immune system and resist various diseases (8).

Tomatoes are rich in *γ*-aminobutyric acid (GABA), a natural non-protein amino acid with the molecular formula C_4_H_9_NO_2_, which is widely found in animals, plants, and microorganisms. In the human body, GABA functions as an inhibitory neurotransmitter and plays a critical role in regulating nervous system activity (9). It has important physiological functions such as lowering blood pressure, reducing stress, and promoting sleep. Studies have shown that GABA supplementation in humans is more effective than medication in alleviating hypertension (10–12). GABA was first discovered in potatoes in 1949 and has since been detected in nearly all major economically important crops (13). The increasing demand for natural GABA has enhanced its commercial value in processed foods (14); therefore, GABA is widely used in food and beverages. In plants, GABA not only helps maintain the carbon-nitrogen balance but also plays a role in responding to oxidative stress, regulating pH, and defending against insects (15–17). Glutamate serves as a precursor of GABA and is converted to GABA by glutamate acid decarboxylase (GAD). This transformation contributes to the unique flavor of tomatoes and other glutamate-rich foods (18, 19).

5-aminolevulinic acid (ALA) is a synthetic precursor of tetrapyrrole biosynthesis and an important intermediate in the production of the photosensitizer protoporphyrin IX (PpIX). It is non-toxic, easily degradable, and contains no residues. In agricultural production, it is used as a plant growth regulator, yield enhancer, herbicide, insecticide, color enhancer, or defoliant (20). As ALA is involved in the regulation of plant growth and development, it is considered a novel growth-regulating substance with various physiological functions (21). Therefore, it has significant potential for agricultural production and offers broad prospects for applications and market development (22). The application of ALA can improve the GABA content in crops. For example, studies have shown that the foliar application of ALA to perennial grass species, such as *Agrostis stolonifera*, increases GABA content in treated plants (23). The exogenous application of ALA to tomato seedlings increased endogenous GABA levels, whereas exogenous GABA application enhanced endogenous ALA levels in the seedlings. ALA and GABA are derived from glutamate (Glu), and both promote plant growth and enhance stress resistance (24). The application of ALA has been shown to improve crop quality. For instance, studies have demonstrated that ALA treatment in peaches (*Amygdalus persica*) significantly increased the content of vitamin C and soluble sugars, enhanced the activities of antioxidant enzymes (SOD, POD, and CAT), and increased the soluble sugar-to-titratable acid ratio. Additionally, it promoted the accumulation of anthocyanins in the peach skin (25). In lettuce (*Lactuca sativa* var. *ramosa* Hort.), exogenous application of ALA increased the content of vitamin C and soluble sugars and reduced nitrate and crude fiber levels, thereby improving its quality and taste (26). Exogenous application of ALA increased the content of soluble solids and proteins in tomatoes by 20.9 and 31.4%, respectively. It also significantly enhanced the sugar content while reducing the total organic acid levels in the tomato fruit. Moreover, ALA application increases the quantity and diversity of volatile compounds during ripening, thereby improving the overall quality of tomato (27, 28). Rhizosphere application of ALA increased the content of ascorbic acid, soluble proteins, soluble solids, and soluble sugars in apple (*Malus domestica* Borkh.) fruit, while reducing titratable acid levels, thereby improving the internal fruit quality (29). Recent studies have shown that root application of ALA to apple trees significantly improved the photosynthetic performance of PS II and PS I in apple leaves. This indicated that ALA-treated apple trees accumulated more assimilates, thereby enhancing fruit quality (30). In recent years, foliar sprayed with ALA (100 mg·L^−1^) solutions during the young fruit’ expansion stage of ‘Shine Muscat’ grapes (*Vitis vinifera* L.) showed a significant increase in single fruit weight, the contents of soluble solids, soluble sugars, and soluble proteins, delayed fruit softening, and improved fruit taste. Measurement of grape fruit aroma components indicated that ALA treatment markedly enhanced the content of alcohols and aldehydes, which are important aroma components in grape fruit (31). Previously, we studied the effects of ALA on tomato (*S. lycopersicum* cv. Yuanwei No.1), during the green ripening stage of fruit maturation. The results showed that treatment with 100 mg·L^−1^ ALA could increase the variety of alcohols, ketones, hydrocarbons, and other volatile substances in the fruit (32).

Given the increasing consumer demand for high-quality fruit nutrition, high GABA content in tomatoes has become an important quality trait. Exogenous ALA, a plant growth regulator, enhances fruit quality, and promotes GABA synthesis and accumulation. However, the mechanisms through which ALA regulates GABA synthesis and accumulation in fruit remain unclear. In this study, the tomato cultivar ‘184’ was used as an experimental material to analyze the composition of nutritional components and GABA synthesis at different application sites and concentrations of ALA. These findings provide a theoretical basis for cultivating high-quality tomatoes and for the practical application of ALA.

## Materials and methods

### Plant materials and treatment

Tomato (*Solanum lycopersicum* cv. ‘184’) seeds were sown on June 21, 2023, and transplanted to a greenhouse on August 3, 2023, when the tomato seedlings developed four true leaves. The tomato plants were cultivated in a trough-based substrate system with drip irrigation using plastic mulch. After the first flower cluster bloomed, the pollination date was recorded. Ten days after pollination, fruit of uniform size at the fruit-setting stage (diameter ≈ 2 cm) were selected. The plants were marked and treated with different concentrations of exogenous ALA using various methods. Treatments were administered every 10 days until maturity. The experimental treatments are presented in Table 1.

**Table 1:** Experimental design for concentration screening treatments.

Treatment type	Treatment names	ALA concentration
Root treatment	CK	0 mg·L^−1^
	ALA-1	10 mg·L^−1^
	ALA-2	50 mg·L^−1^
Leaf treatment	CK	0 mg·L^−1^
	ALA-2	50 mg·L^−1^
	ALA-1	100 mg·L^−1^
Fruit treatment	CK	0 mg·L^−1^
	ALA-1	100 mg·L^−1^
	ALA-2	200 mg·L^−1^

For the ALA treatment as root irrigation, 200 mL of the solution was applied to each plant during each treatment. For the ALA treatment as a foliar spray, a solution containing 0.01% Tween-20 was sprayed until the leaf surface was uniformly covered with droplets. During each treatment, the entire upper and lower surfaces of the four leaves surrounding the fruit clusters were sprayed evenly. The ALA solution sprayed on the fruit surface contained 0.01% Tween-20, and the entire fruit surface was evenly sprayed to ensure that it was covered with droplets. Each treatment was replicated thrice (with three randomly arranged cultivation trays in the greenhouse). To ensure consistency in the effects of ALA treatment, boundary plants were excluded, and six uniformly growing tomato plants were randomly marked in each replicate. Exogenous ALA treatments were conducted at 18:00 (when temperatures drop rapidly in winter in the northwestern region of China) after the greenhouse curtains were closed, followed by 12 h of darkness. All other agronomic management practices were consistent across treatments. During sampling, five mature tomato fruit were randomly selected for each treatment, with three replicates. Samples were immediately brought back to the laboratory for determination of appearance and nutritional quality with the aim of identifying the optimal ALA concentration for root, leaf, and fruit applications. At the fruit-setting stage of the fourth truss of tomato plants, the selected concentrations from the previous screening were applied as follows: root treatments (CK, ALA-1), designated as CK1 and T1; leaf treatments (CK, ALA-2), designated as CK2 and T2; and fruit treatments (CK, ALA-1), designated as CK3 and T3. Treatments were applied every 10 days starting from the fruit-setting stage until the fruit reached maturity. Based on the morphological and color changes observed during fruit development, the dynamic sampling process was categorized into three stages. According to the description by Shinozaki et al. (33), the standard for fruit ripening stages is defined as follows: the mature green stage (full-sized green fruit), the breaker stage (less than 10% green transitioning to orange), the maturity stage (entirely pink fruit), and the red ripe stage (fully red fruit). In this experiment, the mature green stage was observed at 116–123 days after planting, breaker stage at 130–137 days, and full maturity at 144 days.

### Measurement of tomato fruit quality parameters

The single fruit weight was measured using an analytical balance. The soluble sugar content was determined using a method adapted from Grandy et al. (34), with minor modifications. The absorbance of the solution was measured at 620 nm using a UV-1800 ultraviolet–visible spectrophotometer (Shimadzu, Japan) The soluble sugar concentration calculated using the standard curve ranges at 0–160 μg·mL^−1^. Each treatment was replicated thrice, and the average value was calculated. Titratable acidity was determined by grinding 5 g of fresh tomato fruit into a fine paste, which was then transferred to a conical flask. The volume was adjusted to 50 mL using deionized water, and the mixture was filtered. The filtrate was then titrated with 0.1 mol·L^−1^ sodium hydroxide (containing two drops of 1% phenolphthalein) until a faint pink color persisted. The procedure was repeated thrice and the average value was calculated. The sugar-to-acid ratio of tomato fruit was calculated as the ratio of soluble sugar content to organic acid content. The soluble solid content was measured as follows: a sample was cut along the equator, and the juice was squeezed out and placed on the prism surface of a PAL-1 handheld refractometer (ATAGO, Japan) to avoid any seeds. This process was repeated if seeds were present. The sample was allowed to sit for 1 min to ensure uniformity and the absence of bubbles, after which the reading was recorded and expressed as a percentage. The soluble protein content was quantified by homogenizing fresh tomato fruit (0.5 g) in 5 mL of distilled water on ice, followed by centrifugation at 8479 × *g* for 10 min. The supernatant (1 mL) was mixed with Coomassie Brilliant Blue G-250 and incubated for 3 min. Absorbance was measured at 595 nm using a UV-1800 spectrophotometer (Shimadzu, Japan), and the soluble protein concentration was determined based on a standard curve with a concentration range of 0–100 μg·mL^−1^ with each treatment replicated thrice (units: mg·g^−1^ FW). Vitamin C (ASA) content was determined using the 2,6-dichlorophenolindophenol (DCPIP) method. Fresh tomato samples (0.5 g) were homogenized in 1.5 mL of 2% oxalic acid in an ice bath, followed by the addition of 1% oxalic acid to form a homogeneous paste. The mixture was transferred to a 50 mL volumetric flask, rinsed with 1% oxalic acid, and supplemented with 30% zinc sulfate (0.5 mL) and 15% potassium ferrocyanide (0.5 mL). The solution was diluted to 50 mL with 1% oxalic acid, filtered, and the filtrate was collected for analysis. The extract [2 mL of DCPIP solution and xylene (5 mL) were added to 4 mL of the extract]. The solution was shaken and allowed to separate, and the pink upper layer was measured at 500 nm using a UV-1800 spectrophotometer (Shimadzu, Japan). The absorbance was recorded after blanking with xylene. The ascorbic acid concentration was calculated using a standard curve within the range of 0–100 mg·mL^-1^, with each treatment repeated in triplicate.

### Amino acid profile analysis of tomato fruit

Amino acid profile analysis: the preparation of tomato fruit samples and analysis of free amino acid components were conducted according to the method described by Nimbalkar et al. (35), with slight modifications. Twenty amino acid standards were obtained from Merck Millipore (Billerica, MA, United States) and Sigma-Aldrich (St. Louis, MO, United States). For the extraction process, 0.1 g of frozen tomato powder was mixed with 1 mL of 0.5 M hydrochloric acid solution. The mixture was vortexed at 3701 × *g* for 20 min using a vortex mixer (MX-S, Scilogex, San Diego, CA, United States) and then subjected to ultrasonic extraction at 25°C for 20 min (SB-800 DT, NingBo Scientz Biotechnology Co., Ltd., Ningbo, China). After ultrasonication, the sample was centrifuged at 20,000 × *g* for 20 min (3-18KS; Sigma, Osterode am Harz, Germany). The supernatant was filtered through a 0.22 μm aqueous filter, and 5 μL of the filtrate was injected into an HPLC–MS system (LC–MS, Agilent, #1290–6,460, Santa Clara, CA, United States) for quantitative analysis. The HPLC conditions were as follows: an Agilent InfinityLab Poroshell 120 HILIC-Z column (2.1 × 100 mm, 2.7 μm) was used; a 200 mM ammonium formate stock solution was prepared with water and the pH was adjusted to 3 with formic acid. Mobile phase A was composed of water and ammonium formate stock solution (9:1), and mobile phase B consisted of acetonitrile and ammonium formate stock solution (9:1); both mobile phases had a final concentration of 20 mM. The flow rate was set at 0.5 mL·min-1 and the column temperature was maintained at 25°C. The MS conditions were as follows: the ionization mode was ESI positive mode; the drying gas temperature was 330°C, nebulizer pressure was 35 psi, gas flow rate was 13.0 L·min^−1^, sheath gas temperature was 390°C, sheath gas flow rate was 12 L·min^−1^, and capillary voltage was 1,500 V.

### Determination of GABA synthesis-related parameters in tomatoes

The content of glutamate was derived from the free amino acid content determination data described above.

The GABA content was determined using a *γ*-aminobutyric acid (GABA) assay kit (Suzhou Keming Biotechnology Co., Ltd., Suzhou, China), following the manufacturer’s instructions. Each treatment was performed in triplicate, and the average value was calculated. The instructions for the assay kit are as follows. Weigh approximately 0.1 g of fresh tomato sample and add 1 mL of extraction buffer. The mixture was thoroughly homogenized and transferred to an EP tube. Incubate the tube in a 95°C water bath for 2 h, ensuring the cap is tightly sealed to prevent water loss. After cooling, centrifuge at 8,000 × *g* for 10 min at 25°C, and collect the supernatant for analysis. Take 90 μL of the supernatant (for the blank, use 90 μL of extraction buffer) and add 150 μL of Reagent 1 and 120 μL of Reagent 2. The solution was thoroughly mixed and incubated at room temperature for 5 min. Then, add 180 μL of Reagent 3, mix well, and incubate in a 95°C water bath for 10 min. After cooling on ice, add 600 μL of Reagent 4 and mix thoroughly. Then, 1 mL of the mixture was transferred into a 1 mL glass cuvette, and the absorbance was measured at 640 nm. The absorbance values were recorded as samples (*A*_sample_) and blanks (*A*_blank_). The difference in absorbance is calculated as ∆A = *A*_sample_ − *A*_blank_. Finally, calculate the GABA content using the regression equation provided by the kit under standard conditions.

The activity of glutamate acid decarboxylase (GAD) was measured using a plant GAD assay kit (Suzhou Greys Biotech Co., Ltd., Suzhou, China). This procedure was performed according to the manufacturer’s instructions. Each treatment and plant part were analyzed in triplicate, and enzyme activity was calculated based on standard curves.

Total RNA was extracted from tomato fruit using the AG Steady Pure Plant RNA Extraction Kit (Aikeri Biotechnology), and reverse transcription was performed using the AG Evo M-MLV Reverse Transcription Kit (Aikeri Biotechnology). The qRT-PCR reaction system comprised 10 μL, including 0.5 μL of cDNA template, 5 μL of 2 × SYBR Green qPCR Premix (containing ROX Plus) (Aikeri Biotechnology, Beijing, China), and 0.4 μL of each forward and reverse primer, with ddH_2_O added to a final volume of 10 μL. Two-step detection was conducted according to the manufacturer’s instructions and quantification was performed using the comparative CT method (36). The tomato actin gene served as an internal reference, and the primer sequences are listed in Table 2.

**Table 2:** Primer sequences of qRT-PCR.

Gene	Accession number	Forward primer 5′–3’	Reverse primer 5′–3’
*SIGAD1*	NM_001247112.2	AGTGGGTTCATCAGAGGCAATAATG	GCTCCAGTGACTATATTAGGCTTATCG
*SIGAD2*	NM_001246893.1	TGCTGGTATTGGTTGGGTCATTTG	AGAGAAATTGAGGGTGAAAGTAGGTTG
*SIGAD3*	NM_001246898.2	ACTGGCAAAACAAACGCAAAGC	CTCCCAACACACCTGAACATTAGC
*Actin*	Solyc03g078400	TGTCCCTATTTACGAGGGTTATGC	CAGTTAAATCACGACCAGCAAGAT

### Data analysis

Data were analyzed using SPSS 26.0, and GraphPad Prism 10.1.2. Tukey’s HSD test was performed to conduct variance analysis, with a significance level set at *p* < 0.05. Graphical representations were conducted using GraphPad Prism 10.1.2.

## Results

### Effects of different ALA concentration on tomato physiognomic quality

**Figure 1:**
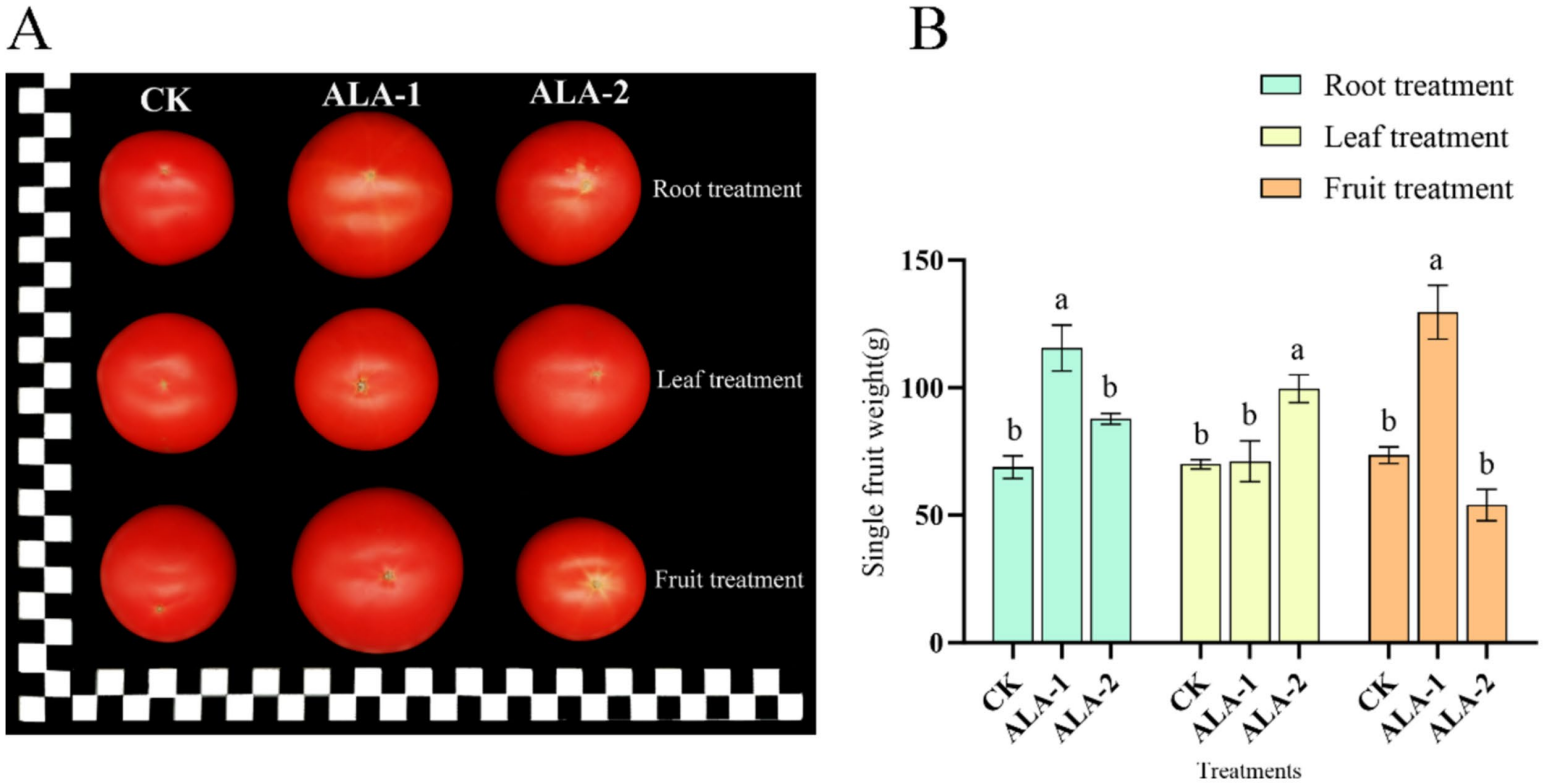
Effect of exogenous ALA application at different sites on single fruit weight and hardness of tomato fruit. **(A)** Photos of tomato the maturity stage (each square of the ruler represents 1 cm). **(B)** Single fruit weight; the short vertical line in the histogram represents the mean ± SE (*n* = 3). The different normal letters indicate that each treatment has significant difference at the 0.05 level.

As shown in Figure 1A, ALA application at all three sites increased tomato fruit size. Among them, the largest fruit weight in each treatment was obtained with root application of 10 mg·L^−1^, foliar application of 100 mg·L^−1^, and fruit application of 100 mg·L^−1^. As shown in Figure 1B, the application of ALA solutions in different ways enhanced the single-fruit weight of tomatoes. Root application of 10 mg·L^−1^ ALA significantly increased the single fruit weight at the mature stage by 67.77% compared to the control. Leaf application of 100 mg·L^−1^ ALA significantly increased the single fruit weight at the mature stage by 42.37% compared to the control. Fruit application of 100 mg·L^−1^ ALA significantly increased the single fruit weight at the mature stage by 76.24% compared to the control.

### Effects of different ALA concentrations on sugar and acid quality of tomato fruit

**Figure 2:**
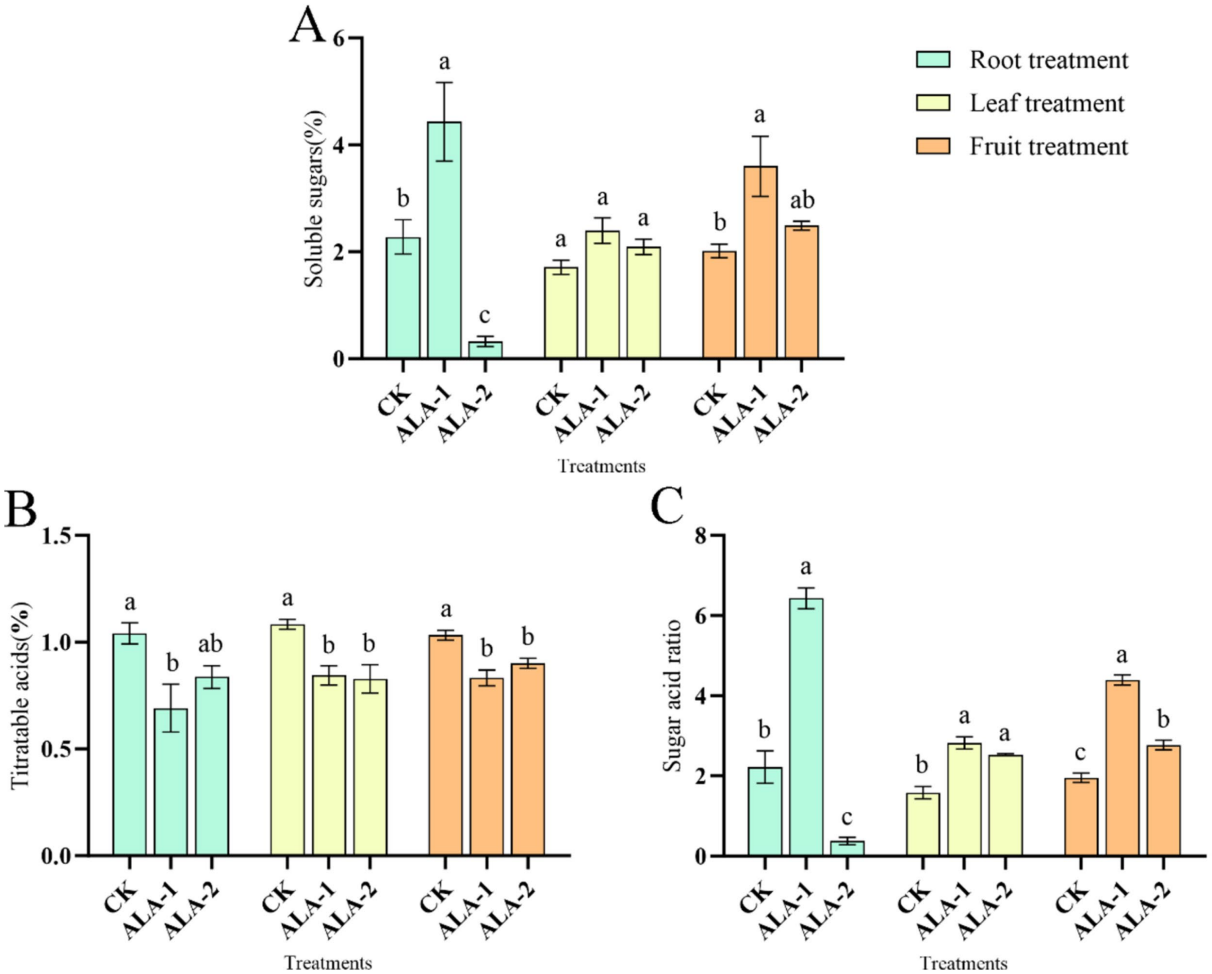
Effects of exogenous ALA on soluble sugar, titrable acid and sugar-acid ratio of tomato fruit. **(A)** Soluble sugar content; **(B)** titrable acid content; **(C)** sugar-acid ratio. The short vertical line in the histogram represents the mean ± SE (*n* = 3). The different normal letters indicate that each treatment has significant difference at the 0.05 level.

The ALA solutions applied to different sites increased the soluble sugar content of tomatoes (Figure 2A). However, root application of 10 mg·L^−1^ and fruit application of 100 mg·L^−1^ ALA significantly increased the soluble sugar content in ripe tomatoes.

Specifically, root application of 10 mg·L^−1^ ALA significantly increased the soluble sugar content in mature tomatoes by 94.52% compared to the control. Leaf application of 50 mg·L^−1^ and 100 mg·L^−1^ ALA also showed higher soluble sugar content than the control, although the differences were not statistically significant. Fruit application of 100 mg·L^−1^ ALA significantly increased the soluble sugar content in mature tomatoes by 78.51% compared to the control.

The ALA solutions at different concentrations applied to different plant sites decreased the titratable acid content in tomato fruit (Figure 2B). Root application of 10 mg·L^−1^ ALA significantly reduced the titratable acid content in mature tomatoes by 33.61% compared to the control. Leaf application of 50 mg·L^−1^ and 100 mg·L^−1^ ALA significantly reduced the titratable acid content in mature tomato peels by 22.05 and 23.62%, respectively. The application of 100 mg·L^−1^ and 200 mg·L^−1^ ALA significantly reduced the titratable acid content in mature tomatoes by 19.42 and 12.81%, respectively.

The ALA solutions applied to different sites increased the sugar-acid ratio in tomatoes (Figure 2C). However, root application of 10 mg·L^−1^, foliar application of 50 mg·L^−1^ and 100 mg·L^−1^, and fruit application of 100 mg·L^−1^ and 200 mg·L^−1^ ALA significantly increased the sugar-to-acid ratio in ripe tomatoes. In contrast, root application of 50 mg·L^−1^ ALA reduced the sugar-to-acid ratio, while other ALA treatment concentrations had no effect on this parameter. Root application of 10 mg·L^−1^ ALA significantly increased the sugar-acid ratio in mature tomatoes by 1.90 times compared to the control. Leaf application of 50 mg·L^−1^ and 100 mg·L^−1^ ALA significantly increased the sugar-acid ratio in mature tomatoes by 0.78 times and 0.60 times, respectively. Fruit application of 100 mg·L^−1^ ALA and 200 mg·L^−1^ ALA significantly increased the sugar-acid ratio in mature tomatoes by 1.25 times and 0.42 times, respectively, compared to the control.

### Effects of different ALA concentrations on soluble solids, soluble protein, and ascorbic acid content in tomato fruit

**Figure 3:**
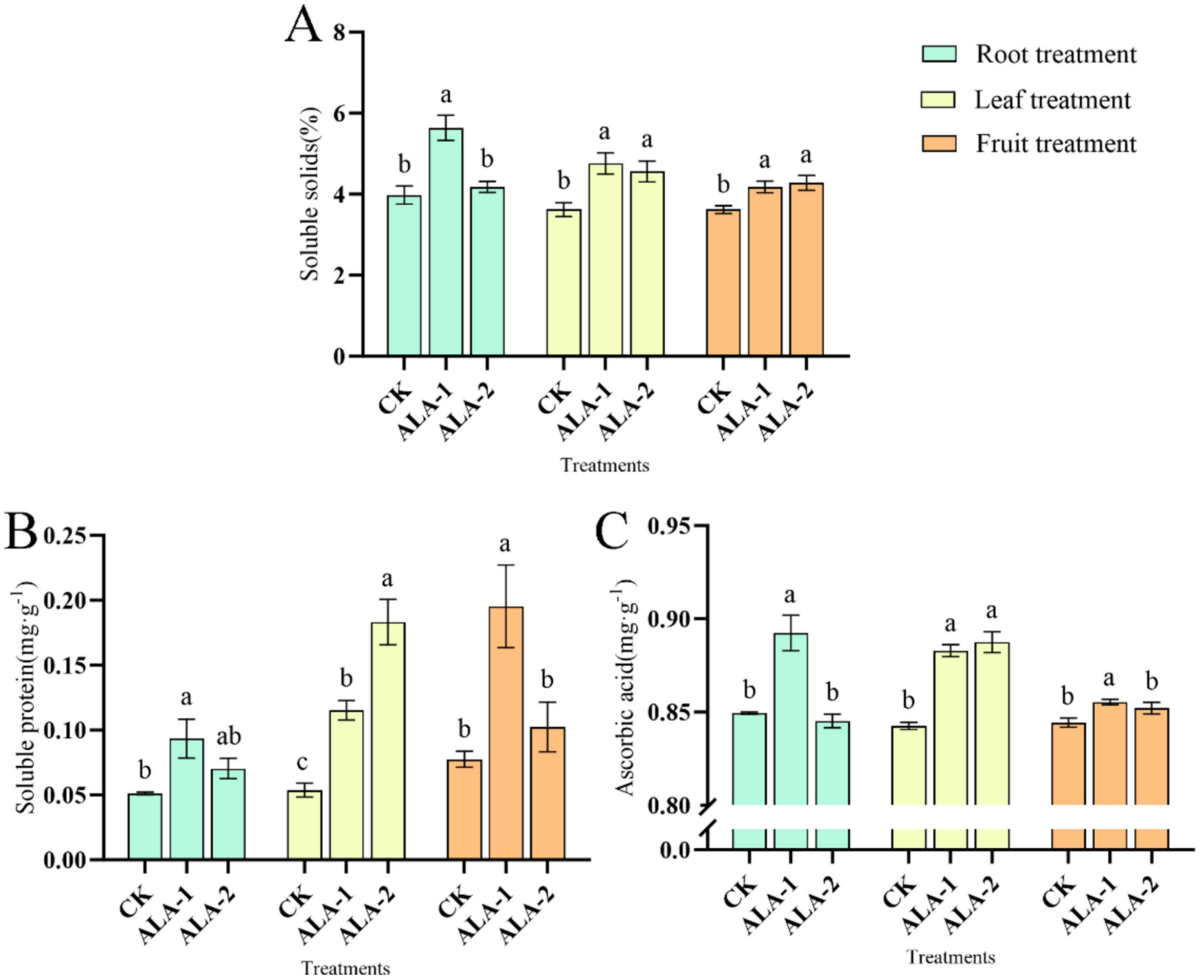
Effects of exogenous ALA applied at different sites on soluble solids, soluble proteins, and acid in tomato fruit. **(A)** Soluble Solids content; **(B)** soluble protein content; **(C)** ascorbic acid content. The short vertical line in the histogram represents the mean ± SE (*n* = 3). The different normal letters indicate that each treatment has significant difference at the 0.05 level.

The ALA solutions applied to different plant sites enhanced the soluble solid content of tomatoes (Figure 3A). Specifically, root application of 10 mg·L^−1^ ALA significantly increased the soluble solids content in mature tomatoes by 41.71% compared to the control. Leaf application of 50 mg·L^−1^ and 100 mg·L^−1^ ALA significantly increased the soluble solids content in mature tomatoes by 13.88 and 9.09%, respectively. Fruit application of 100 mg·L^−1^ and 200 mg·L^−1^ ALA significantly increased the soluble solids content in mature tomatoes by 15.50 and16.67%, respectively, compared to the control. Figure 3B shows that ALA solutions applied to different plant sites enhanced the soluble protein content in tomatoes. Application of 10 mg·L^−1^ ALA irrigated on root significantly increased the soluble protein content in mature tomatoes by 82.71% compared to the control. Leaf application of 50 mg·L^−1^ and 100 mg·L^−1^ ALA significantly increased the soluble protein content in mature tomatoes by 114.71 and 241.05%, respectively, compared to the control. Fruit application of 100 mg·L^−1^ ALA increased the soluble protein content in mature tomatoes by 152.32% compared to the control.

Exogenous ALA solutions applied to different plant sites enhanced ascorbic acid content in tomatoes (Figure 3C). However, root application of 10 mg·L^−1^, foliar application of 50 mg·L^−1^ and 100 mg·L^−1^, and fruit application of 100 mg·L^−1^ ALA significantly increased the ascorbic acid content in ripe tomatoes, whereas other ALA treatment concentrations had no effect on ascorbic acid content. Root application of 10 mg·L^−1^ ALA significantly increased the ascorbic acid content in mature tomatoes by 5.06% compared to the control. Leaf application of 50 mg·L^−1^ and 100 mg·L^−1^ ALA significantly increased the ascorbic acid content in mature tomatoes by 4.79 and 5.33%, respectively. Fruit application of 100 mg·L^−1^ ALA significantly increased the ascorbic acid content in mature tomatoes by 1.31% compared to the control.

### Effect of exogenous ALA on free amino acid composition in tomato fruit

**Figure 4:**
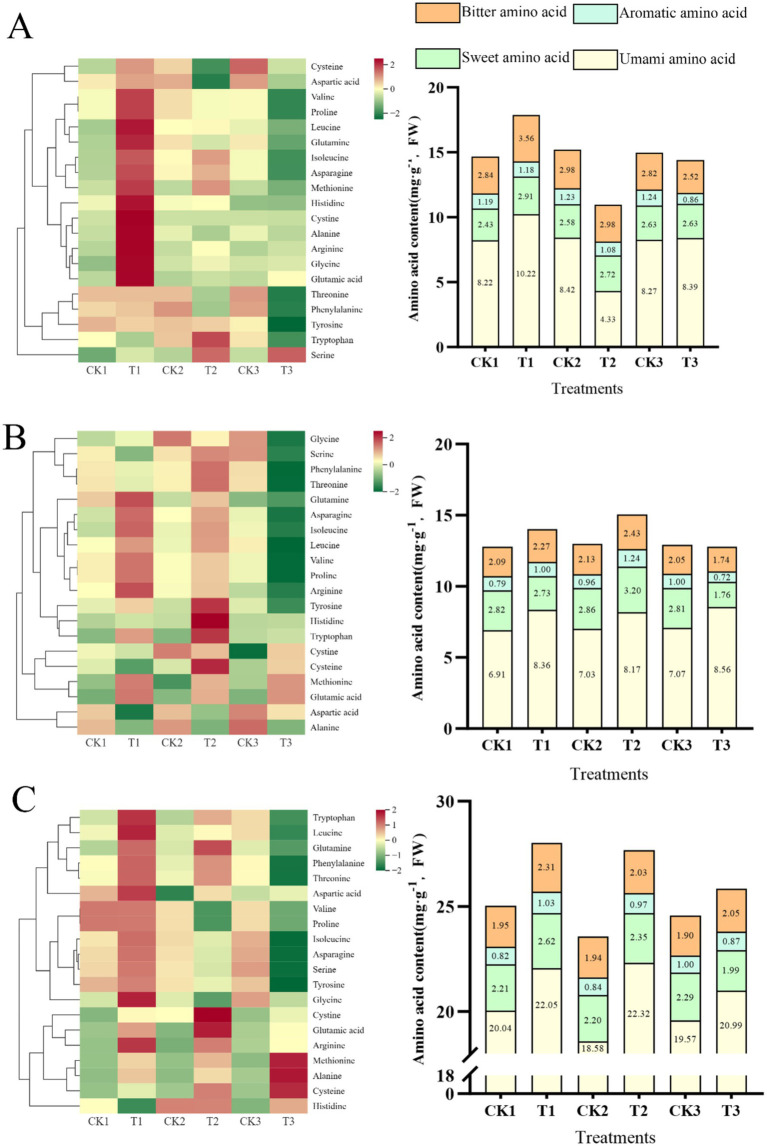
Effect of exogenous ALA application to different parts on amino acid composition in tomato fruit **(A)** mature green stage amino acid content; **(B)** breaker stage amino acid content; **(C)** maturity stage amino acid content.

Figure 4 illustrates the effect of exogenous ALA application to various plant parts on the free amino acid composition of tomato. In the present study, 20 free amino acids, including both essential and non-essential amino acids, were detected during the three ripening stages. The essential amino acids in humans include phenylalanine, methionine, isoleucine, tryptophan, valine, leucine, histidine, and threonine. Non-essential amino acids include glutamic acid, cysteine, arginine, glycine, serine, proline, tyrosine, alanine, cystine, aspartic acid, asparagine, and glutamine. Based on taste properties, these 20 amino acids were categorized into four groups: umami (glutamic and aspartic acids), bitter (arginine, histidine, leucine, tyrosine, valine, isoleucine, and phenylalanine), sweet (proline, alanine, threonine, glycine, serine, and asparagine), and aromatic (phenylalanine, tyrosine, and tryptophan). The levels of these amino acids, particularly umami, increased significantly during fruit maturation.

During the mature green stage of the fruit, the total amino acid content was 21.08% higher with root-applied exogenous ALA than with the other treatments (Figure 4A). The umami amino acid content was also higher (24.33%) than that of the control.

During the breaker stage of the fruit, the total amino acid content was higher with root and leaf ALA applications than in the control groups, with increases of 8.39 and 18.08%, respectively (Figure 4B). Umami amino acids were higher in the root, leaf, and fruit treated with exogenous ALA than in their respective control groups, with increases of 35.06, 16.22, and 21.07%, respectively.

In addition, during the mature stage, the total amino acid content with root and leaf applications was higher than that in the control, with increases of 11.22 and 16.5%, respectively (Figure 4C). The umami amino acid content in fruit treated with roots, leaves, and fruit was higher than that in their respective controls, with increases of 10.03, 20.13, and 7.26%, respectively.

### The effects of exogenous ALA on GABA synthesis in tomato fruit

**Figure 5:**
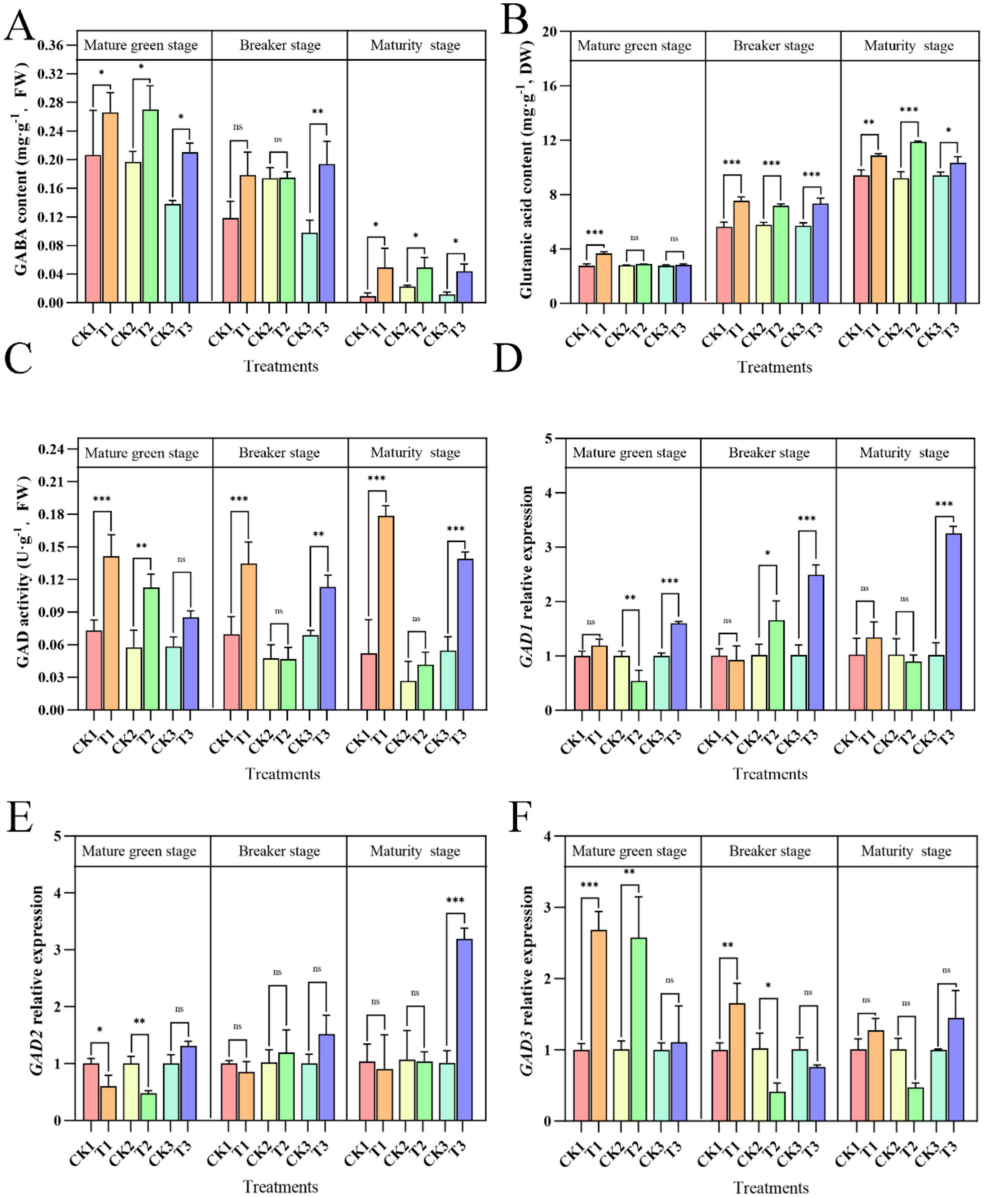
Effects of exogenous ALA on GABA biosynthesis indexes in tomato fruit. Asterisks indicate significance levels for Tukey’s HSD test between different application sites at the same time point, with *p < 0.05, **p < 0.01, ***p < 0.001, and ns indicating no significant difference. **(A)** GABA content; **(B)** Glutamate acid content; **(C)** GAD activity; **(D)** *GAD1* Relative expression; **(E)** *GAD2* Relative expression **(F)** *GAD3* Relative expression.

As shown in Figure 5A, the GABA content in tomato fruit was lower at the mature stage than at other stages, indicating that the GABA content in the fruit decreased during the ripening process. However, exogenous ALA application to different parts of the plant increased GABA content in the fruit. During the mature green stage, exogenous ALA applied to the roots, leaves, or fruit significantly increased the GABA content in tomato fruit, with increases of 45.16, 41.83, and 32.54%, respectively, compared with the control groups. In the breaker stage, fruit-applied exogenous ALA significantly increased the GABA content in tomato fruit by 98.41% compared to the control. Although root and leaf applications of ALA showed numerical increases compared to the controls during the maturity stage, these increases were not statistically significant. During the mature stage, the GABA content in tomato fruit decreases rapidly, but exogenous ALA application to the roots, leaves, or fruit effectively slows this decline. Compared to the control, ALA applied to different parts of the plant significantly increased the GABA content in tomato fruit by 433.32, 214.58, and 373.98%, respectively.

The glutamate content increased as the tomatoes matured (Figure 5B). During the mature-green stage, the application of exogenous ALA to plant roots significantly increased the glutamate content in tomato fruit, showing an increase of 32.50% compared with that of the control. However, foliar and fruit applications only showed numerical increases without significant differences compared with the control. During the breaker stage, exogenous ALA applied to different parts significantly increased glutamate content in tomatoes compared to that in the control groups, in which the root treatment improved glutamate content by 33.32%, leaf treatment by 24.25%, and fruit treatment by 28.57%. At the maturity stage, exogenous ALA applied to different parts significantly increased the glutamate content in tomatoes by 15.60, 28.73, and 9.96% in the root, leaf, and fruit treatments, respectively.

In the control and leaf ALA groups, GAD activity decreased as the tomatoes matured (Figure 5C). However, root- and fruit-applied exogenous ALA increased GAD activity as the tomatoes matured. Exogenous ALA applied to different plant parts can enhance GAD activity in tomato plants. In the mature green stage, root and leaf application of exogenous ALA significantly increased GAD activity in tomato fruit by 90.30 and 94.31%, respectively, compared with the control, whereas fruit application showed a numerical increase without a significant difference. In the breaker stage, root and fruit applications of exogenous ALA significantly increased GAD activity by 93.00 and 63.88%, respectively, compared to the control, whereas leaf application showed a numerical increase without a significant difference. At the maturity stage, root and fruit application of exogenous ALA significantly increased GAD activity in tomato fruit by 241.17 and 154.19%, respectively, compared with the control, whereas leaf application showed a numerical increase without a significant difference. As shown in Figures 5D–F, root application of ALA during the mature-green stage significantly increased the relative expression of *GAD3* in tomatoes by 167.45% compared to the control. Leaf application of ALA also significantly enhanced the relative expression of *GAD3*, increasing it by 156.22% compared with the control. Furthermore, fruit application of ALA significantly elevated the relative expression of *GAD1* by 60.15% compared with the control. During the breaker stage, root application of ALA significantly increased the relative expression of *GAD3* by 64.89% compared with the control. Leaf application of ALA notably enhanced the relative expression of *GAD1*, increasing it by 63.75% compared with the control. Additionally, fruit application of ALA significantly increased the relative expression of *GAD1* by 146.24% compared with the control. In the mature stage, fruit application of ALA significantly boosted the relative expression of *GAD1* and *GAD2*, resulting in increases of 219.37 and 214.26%, respectively, compared to the control.

## Discussion

Aminolevulinic acid affects chlorophyll synthesis in crop leaves and enhances the photosynthetic capacity, thereby promoting plant growth and development. At appropriate concentrations, it acts as a plant growth regulator by modulating various physiological activities (37). This study indicated that the application of ALA solutions at different plant sites, such as the roots, leaves, and fruit surfaces, can enhance the single fruit weight of tomatoes. This finding is consistent with previous research showing that ALA application, through root drenching, foliar spraying, or fruit surface spraying, can enhance tomato yield (30). Similarly, ALA applied to apple plants improved single-fruit weight under both root drenching and foliar application (38). Soluble solids are critical indicators of fruit quality and primarily consist of soluble sugars, organic acids, and other nutritional components. Soluble solids are a critical indicator of tomato quality and a key factor that influences both tomato quality and consumer preference. They account for more than 60% of the dry matter of tomatoes and are primarily composed of soluble sugars, organic acids, mineral elements, and other nutrients. Soluble sugars and organic acids in tomato fruit play a particularly important role in determining the overall quality (8, 39, 40). In this study, root irrigation (10 mg·L^−1^ALA), as well as leaf and fruit surface application (100 mg·L^−1^ ALA) significantly increased the soluble sugar content in tomato fruit. All treatments with appropriate ALA concentrations significantly reduced the organic acid content in tomato fruit, which subsequently improved the soluble solid content and sugar acid ratio of the tomatoes. This is consistent with previous research which demonstrated that a concentration of 300 mg·L^−1^ ALA could enhance the soluble sugar content in ‘Rabbit Eye’ blueberries (*Vaccinium uliginosum*) (41). Previous research on grape fruit has found that treatment with 100 mg·L^−1^ ALA effectively reduced acidity of fruit, the content of soluble solid substances in the fruit increased by 2.7% (42). Previous studies have shown that foliar application of ALA to blueberry plants can reduce the titratable acid content of blueberry fruit (43). Soluble protein and Vc contents in plants are crucial physiological indicators of vegetable quality and nutritional value. In this study, the application of exogenous ALA to the root, leaf, or fruit surface significantly increased soluble protein and ascorbic acid content in tomatoes. Foliage sprayed with ALA significantly enhanced the levels of soluble proteins and Vc in apples (38). These findings indicate that applying exogenous ALA to plants can improve the nutritional quality of tomato fruit, regardless of the application.

Amino acids are crucial active compounds in living organisms, and their content is a key indicator of food quality and nutritional value (44). As one of the essential bioactive components of fruit, amino acids significantly influence the nutritional value, aroma, taste, and health benefits of fruit. However, the functions of the different amino acids vary (45). Amino acids also possess distinct taste characteristics such as sweetness, bitterness, and umami, and their levels play a crucial role in shaping the flavor quality of fruit (46). The results of this study indicate that the content of various taste-related amino acids in tomatoes significantly increases during maturation. Additionally, the application of exogenous ALA at all three treatment locations notably enhanced free amino acid content. Studies have shown that the application of ALA to tomato fruit increases the total amino acid content (40). The enhancement of umami amino acids by exogenous ALA is particularly pronounced, with glutamic acid being the primary umami amino acid (47). Glutamic acid (Glu), a precursor of both GABA and ALA, plays a crucial role in plant GABA in plants (19, 48). In this study, the glutamic acid content in tomatoes increased gradually as the fruit matured, reaching its peak at the mature stage across all treatments. During the breaker and maturity stages, application of ALA at all three plant sites significantly enhanced the glutamic acid content in tomato fruit compared to the control. Ammonium is initially converted into glutamine by Glutamine Synthetase (GS) and then to glutamic acid by Glutamate Synthase (GOGAT), thereby increasing the glutamic acid content (49). Studies have shown that Exogenous ALA promotes nitrate reduction and ammonium assimilation in watermelon plants under salt stress conditions.

Tomatoes are one of the vegetables with the highest GABA content among all vegetables (14). GABA helps relax overly active brain cells, significantly reducing the excitability of nerve cells, and thereby improving sleep quality. Additionally, GABA promotes brain metabolism to enhance memory, has diuretic effects, boosts immunity, and helps prevent obesity, among other beneficial physiological functions (50–53). In the present study, the GABA content in tomatoes decreased as the fruit matured, with the most rapid decline occurring between the breaker and maturity stages. However, at maturity, the GABA content in tomatoes treated with exogenous ALA at all three application sites was significantly higher than that in the control. Application of ALA at the three sites effectively slowed the decline in GABA levels. It has been proved that under low-temperature stress, exogenous ALA enhanced GABA synthesis in tomato seedlings. Exogenous ALA can be directly absorbed by plants and promotes the conversion of Glu to GABA through internal feedback mechanisms (24). Thus, it can be inferred that the application of ALA at the three different locations in tomato fruit promotes the conversion of Glu to GABA. In plants, Glu is decarboxylated to produce GABA under the catalysis of glutamate decarboxylase (GAD), a key enzyme involved in this process (19). In a previous study, GAD activity was shown to decrease during tomato maturation. However, ALA enhanced GAD activity and effectively delayed this decline. At the green maturity stage, both root and leaf applications of ALA significantly increased GAD activity. In the breaker stage, fruit application of ALA notably enhanced GAD activity. In the mature stage, GAD activities in both the root and fruit treatments were significantly enhanced compared to the mature stage in the control group. During tomato fruit development, especially from the mature green stage to the mature stage, the expression levels of *GAD1*, *GAD2* and *GAD3* were upregulated by ALA treatment at different plant sites to different extents. ALA applied to different parts of the plant at various stages of tomato maturation enhanced the expression of these three key genes affecting GAD activity, thereby increasing GAD activity and promoting GABA accumulation. Previous research has shown that methyl jasmonate treatment promotes an increase in GAD activity and GABA content in loquat (*Eriobotrya japonica*) fruit, thereby reducing the occurrence of chilling injury (54). Our results are similar to those of previous studies. These results indicate that exogenous ALA can improve GABA biosynthesis and favor fruit quality establishment in tomatoes.

## Conclusion

This study demonstrated that applying exogenous ALA at the optimal concentration to different parts of tomato plants (roots, leaves, and fruit) significantly improved fruit quality, including single fruit weight, soluble solids, soluble protein, ascorbic acid, and sugar-acid ratio in tomatoes. In terms of nutritional quality, ALA notably increased the free amino acid content. Additionally, GABA content was increased by ALA treatment by upregulating the relative gene expression (*GAD1*, *GAD2*, and *GAD3*) and enhancing GAD activity. In summary, exogenous ALA application at different plant sites can improve the flavor and nutritional quality of tomato fruit.

## Data Availability

The original contributions presented in the study are included in the article. Further inquiries can be directed to the corresponding authors.
